# Learning from tinnitus patients' narratives—A case study in the psychodynamic approach

**DOI:** 10.3402/qhw.v7i0.19540

**Published:** 2012-12-27

**Authors:** Nicolas Dauman, Soly I. Erlandsson

**Affiliations:** 1 Département de psychologie, Université de Poitiers, Poitiers, France; 2 Department of Social and Behavioural Studies, University West, Trollhättan, Sweden

**Keywords:** Tinnitus suffering, emotional drives, over-determination, personal history, authentic listening, psychodynamic insight

## Abstract

Tinnitus is assumed to be the perception of sound that results exclusively from activity within the nervous system without any external stimulation. Approximately 1–2% of the population regard their tinnitus as a serious threat towards their quality of life. The way the patients describe their suffering varies, sometimes also depending on the interest and insight of the clinician to whom they turn to for help. The lack of insightful narratives of someone who is severely annoyed by the presence of a constant tinnitus sound may lead to limited and biased models of tinnitus suffering. In the present case study the participating patient, a woman aged 70, shared her experience of being victimized by tinnitus with the clinician/researcher during a number of psychotherapeutic sessions. The psychodynamic, narrative approach, made it possible for the client to articulate the unique and specific meaning that she experienced as being part of her suffering. In her words, tinnitus became a tolerable symptom that she managed to work through within psychotherapeutic alliance.

Tinnitus, according to literature within the neurological field, is assumed to "be the perception of sound that results exclusively from activity within the nervous system without any corresponding mechanical, vibratory activity within the cochlea, and not related to external stimulation of any kind" (Jastreboff & Hazell, 2004). Patients often describe their tinnitus as buzzing or ringing sounds that can increase in loudness and change in pitch, sometimes day-by-day (Stouffer & Tyler, 1990). Epidemiological studies have shown that approximately 8% of the population regard their tinnitus as somewhat disturbing and 1–2% of cases regard it as a serious threat towards quality of life. Sounds can become intrusive when someone who finds them unbearable is forced to listen to them as in the case of an annoying tinnitus that demands constant attention. When tinnitus makes it impossible to live your life the way you feel would be normal, it signifies an important psychological message.

As a symptom, tinnitus has a long history, dated as far back as the seventh century B.C. according to Stephens (1984, 2000). Stephens made a thorough review of the history of tinnitus and described a number of odd treatments being used for its alleviation. One type of tinnitus, described in an ancient Babylonian clay tablet, was the "singing" tinnitus, which was regarded as stemming from someone being seized by a ghost. In Islamic and medieval medicine, tinnitus was described as chronic noises arising from a special form of humours (thick and viscid) and it was also associated with heightened sensibility, for example, phono-phobia. Itard, at the beginning of the 19th century (1821), made a categorization of tinnitus and his two major categories were named "true" (an objective form) and "false" tinnitus (without any acoustical basis). In the latter, he included another type—"fantastic" tinnitus referred to as auditory hallucinations. According to Stephens (2000), Itard represents a more modern approach to tinnitus, as his orientation in trying to manage the problem was directed towards the consequences rather than to the tinnitus itself. Itard's approach has withstood the test of time partly due to failure to treat tinnitus with successful outcomes; that is, that tinnitus disappears altogether. The patients can sometimes hardly see or accept any other solution than becoming totally free from tinnitus and, with such expectation of a complete cure, the risk for frustration is likely to be elevated.

Tinnitus and its association with different acoustic and audiologic parameters— among them hearing impairment and hypersensitivity to noise—has generally been regarded as the most pertinent focus within the research. Reasons for such a focus might rest on attempts to gain better knowledge about the acoustic and physical mechanisms of tinnitus. Studies undertaken by the use of PET-scan (positron emissions tomography) have resulted in certain registrations of what the researchers believe is an increased activity in the hearing-centre of the brain in people with tinnitus (Mirz et al., 1999). An abnormal association between the limbic system and the hearing-centre has also been observed. This finding points to what psychology has long argued; that is, that the perception of tinnitus is related to cognitive and emotional reactions situated in the limbic system. Therefore, in addition to considering tinnitus as a complication following a hearing impairment or a sudden noise trauma, it is essential to pay special attention to the individual patients and their need to recognize the source of their complaints (Erlandsson, 1998). Simply listen to the story that is told by a tinnitus sufferer. Explanations to the emotional suffering of the patient may be linked to personal experiences in life and early childhood. A traumatic experience that has been repressed for a long time can manifest itself in the wake of tinnitus onset (Erlandsson, 2000).

The way patients describe their suffering varies, sometimes also depending on the interest and insight of the person/clinician to whom they turn to for help. Too often the encounter with the care-taking unit will lead to disappointment, sometimes with both parties, that is, the help-seeker and the care-taker. To avoid such a distressing outcome, the care-taking party must be confident enough to accept her or his limited knowledge of the patient's inner conflict. In a person who suffers from anxiety and tinnitus, the emotional motives behind their complaints are structured according to unconscious logic (Holloway & Jefferson, 2001). The lack of insightful and deep narratives of those who are severely annoyed by the presence of a constant tinnitus sound may lead to limited and biased models of patients' complaints. Edgren-Sundin (1993) showed in a qualitative pilot study that participating subjects needed to grieve the loss of silence after the debut of tinnitus. As there are very few formal procedures to help the person to manage a loss of this kind, the burden can hardly be shared with anyone. Some of the participants, in the study by Edgren-Sundin, had been under an increased level of emotional stress before the onset of tinnitus. As they were highly strained when tinnitus occurred, their psychological defences against the critical incidence were also weakened. Someone who experiences an increased and prolonged level of stress has little tolerance for even a faint sound appearing in the ear or in the head (Erlandsson, 2008). This kind of associative incidence might be more prevalent than what research so far has shown.

There are very few, if any, illuminating case studies found in the literature on tinnitus in which the patient's narrative is the main focus for research. Tinnitus, as a symptom, is a common pathway for several underlying disorders and conditions, for example, hypertension, heart disease, as well as different stress-related conditions. Hence, studies investigating the perspective of the individual patient could contribute to an enhanced insight into the circumstances leading to tinnitus suffering. In the present case study, the participating patient, a woman aged 70, has shared her experiences of being victimized by tinnitus with the researcher/clinician through a number of psychotherapeutic sessions. According to Spense (1982), the narrative tradition in psychotherapy dates back to Freud.

## Aims and related research questions

Although some clinical psychologists see tinnitus patients as psychotherapists and offer counselling in a humanistic approach (Erlandsson, 1998; Mohr, 2008), the most influential streams in psychology are not being represented in the literature on the management of tinnitus suffering. This is especially true for the psychodynamic approach, the last contributions referring to this scope on tinnitus dating back to the 1950s (Schneer, 1956; Weinshel, 1955). One aim of our research is to start a collective reflexion about tinnitus suffering from a psychodynamic contemporary scope, enhancing a clinical practice in its methodological and theoretical background. There is also a need to offer a diversity of psychological approaches because of the diversity of tinnitus patients' personalities and motivations. Our starting point was to answer the following question: how to inform clinicians about the psychodynamic approach of tinnitus suffering? It appears that the best way would be to present a single case study, referring to the patient's narrative and how it develops during the course of psychotherapy. By the use of a psychodynamic, narrative approach, the unique and specific meanings experienced by an individual patient caught in a situation characterized by fear can be articulated. The main aim of this approach is to capture the subjective nature of self in its full complexity (Crossley, 2000).

## Method

### Subject and procedure

The participant was a 70-year old woman who consulted a tinnitus clinic for a severe and annoying tinnitus that she, from a psychological viewpoint, experienced as a life-threatening symptom. Part of her story includes intrusive thoughts, anxiety, and suicidal thoughts. However, there was no suicidal attempt. After the consultation with a physician specializing in audiology, the patient was referred to a psychologist and offered psychotherapy. The patient, who lived far from the hospital, could not afford the traveling costs from her home to the hospital; therefore, the psychotherapy was carried out by telephone. In total, 16 psychotherapy sessions were performed over a period of 8 months. Each session, except one, lasted for about half an hour. The penultimate session took place in the patient's home and lasted one and a half hours. After each session the psychotherapist recalled and wrote down the dialogues with the patient and his immediate reflections and interpretations of what the patient had disclosed. After the therapy sessions ended, the psychotherapist made a thorough review of the psychotherapy process with reference to the literature.

### The psychodynamic therapeutic approach

The psychodynamic psychotherapy method stands on two correlative dimensions, that is, the attitude of the patient and the therapist during the therapeutic process. The acceptance of a free and spontaneous discourse from the patient about her present situation has its therapist's counterpart. Freud recommended that the clinician should not take notes during the session with the client, emphasizing that the benefit in precision does not justify the resulting poorer attention to the patient's singular discourse. While this discourse can be recorded today, allowing the clinician to be more available to the patient's narrative, it remains a choice for the psychotherapist involved in the scientific research to do so or not. Recording a psychotherapy session is not a neutral condition for a suffering patient, especially when her suffering is not obvious to others as in the case of tinnitus. In this subjective condition, the need of being trusted by the therapist and endured confidentiality is mandatory throughout the therapeutic process. The client must be confident about the authenticity of the therapist's listening. Hence, recording the sessions can be perceived as a competitive project that the clinicians pursue, for which the emotional condition of the patient takes a back seat. It was therefore decided not to make tape recordings during the psychotherapeutic sessions. Instead, the therapist made notes after each session of the memories that came spontaneously in a discourse form. These recollected notes were structured into a comprehensive narrative including quotations from the patient's singular discourse. Finally, the case history was constructed as a retrospective narrative, from a medical complaint to a traumatic incident for which theoretical reflections were written down in order to enlighten the progress of the patient's narrative.

### The narrative interview method

According to Sarbin (1986), personal narrative has a temporal dimension (a beginning, a middle, and an end) that is held together by a pattern of events, named as a plot. Gergen and Gergen (1983) have classified the plot that people use in making sense of negative events, such as suffering from serious illnesses into three main dimensions: stability, progression, and regression. Crossley (2000) describes the narrative principle as a humanistic image of self as a teller of stories, and as Sarbin (ibid) puts it, we can reflect on any slice of life, for example, hopes, dreams, fears, and fantasies as well as daily routines. The psychoanalytic interview with its recognition of the importance of the unconscious can be used as a model for qualitative research (Kvale, 1986). According to Holloway and Jefferson (2001), the narrative interview method building on free association is the best way to grasp the meanings underlying interviewees' evoked narratives. By using free associations that respect the patients' Gestalt, the narrative that is not structured according to conscious logic can be elicited. We used the narrative method at three different stages of this research: (a) during the psychotherapeutic process, because the patient was invited to talk about her life history and her feelings regarding the symptoms; (b) during the recollection of data from a spontaneous and discursive form to a narrative of each session; (c) when the psychotherapeutic process was synthesized into a case history.

### Ethical considerations

Informed consent was given by the patient to publish parts of the psychotherapy notes, after being informed of our wish to use it for the research on tinnitus suffering. She was told that any publication of the study would be addressed to her. The patient explicitly asked to have that kind of information as she considered her case to be a contribution to the research on tinnitus. The research has been carried out following the principles of the Declaration of Helsinki (1975; revised 2008)—a statement of ethical principles to provide guidance in research involving human subjects. The study was approved by the Ethical committee CPP du Sud-Ouest et Outre-mer III (People Protection Committee); Protocol ref. CPP: DC 2012/95.

## Results

In order to present the case clearly, the use of labels for the main parts allowed us to gather the most significant clinical data regarding the therapeutic process. The four labels, given below, evolved out of the interview material with a focus on an individual life story that disclosed a deeper suffering behind the complaint of tinnitus. Each label gathers a viewpoint on the patient's discourse from a descriptive perspective, that is, part one and two to a theoretical perspective on the disclosing progress, that is, part three and four. The four labels are: Complaints about tinnitus; Beyond the medical condition; Narrative breakdown and psychotherapy; Psychodynamic insights on emotional drives.

### Complaints about tinnitus

Lucie lives alone in an apartment that she feels is too big for her. She divorced more than 40 years ago and decided not to marry again. She has a daughter and a son who do not visit very often. They have their own families. Lucie used to feel comfortable being alone in her home, but during the last couple of months the loneliness has become a burden to her. She feels tired and depressed but does not know why, and has no explanation for her present uncomfortable situation. At her first appointment in the tinnitus clinic, she started to cry and apologized for her behaviour. She explained that she always had felt that she was a strong person managing life on her own. The way she is reacting when having difficulties now is new to her. Driven by despair, she even wanted to throw herself from a bridge *to end this tinnitus*. But the thoughts of her grandson who she feels needs her is holding her back.

It seems hard for Lucie to describe the tinnitus she hears: *It is difficult to say what it sounds like, it is really difficult to describe it*. She believes that there are three different sounds, sometimes in one side more than in the other, sometimes all three inside her head. First of all, she hears a strong blowing *like the wind*; then a sort of crackling sound that she can only mimic as the sound *zzzzz* …; and finally a lapping *toc, toc* …. The description Lucie gives of her tinnitus changes during the months of the psychotherapeutic sessions: *It makes me hear like there are bees around my head*, she explained mimicking another sound *vvvv … Sometimes they are really too much, I can't bear them when there is noise around me*. Lucie remembers her reaction when tinnitus first occurred. She was just coming back from 2 weeks of hospitalization: *I heard a sound in my apartment. I thought it was the electric fan. I called my daughter to see if she heard the same thing as me*. But her daughter could not hear the sound. Lucie had to face noises that she thought were from upstairs before she was admitted to hospital for depression. Neither the neighbour nor the caretaker (concierge) could hear the noises and the tinnitus. One day she called the firemen and the police because of all the noises she thought she heard from upstairs. The noises frightened her. She was so exhausted that she went out during the cold to wait for hours for the noises to stop. When she came back to the apartment the noises upstairs stopped followed by a few days of silence again. But the tinnitus suddenly begun to annoy her again *and they did not leave me alone* she said.

Lucie remembers her fear when tinnitus began to disturb her, and all the questions that followed during many nights. She walked around in the apartment for hours: *What is it? Where does it come from? What have I done to have that in my ears all day long?* For a long time after she consulted her family doctor she searched for answers about her tinnitus. Her doctor did not really comfort her: *You have tinnitus, Madam*. She remembered what he said. *You will have it until the end of your days. I did not understand, what does it mean, 'tinnitus'? So he said to me: it is your ears, they are worn out*. Her psychiatrist told her that she was depressed. She wondered if the drugs she was given had *wrecked her ears*. She noticed odd things around her apartment, and she wondered if: *a third radio relay was set up near her flat, a few times before the tinnitus onset*. She thought that maybe the Hertzian waves of the radio relay have done something to her ears. Nobody believed her and did not take her suspicions seriously, either about her worries from the noises upstairs nor the radio sound waves. Her daughter told her that it was the nerves, and that she was spending too many hours alone at home. Her son explained his view in even harsher words: she was unable to manage by herself. He was furious, because Lucie refused to move to a smaller apartment that they had found in a nursing home for the elderly.

Coming back from the hospital to the apartment was a disaster for Lucie.

When the tinnitus began I couldn't even sleep, I heard it all the time … I was only able to walk and walk again during the night, I couldn't stop walking, to bear it … The onset is awful, one cannot stand it.

Lucie remembers the first remedies she found against the crisis.

After several months she managed to "habituate to it", like people say. Now I pay less attention to the tinnitus, I do not put the hairdryer on my ears any more … I did that for days and days … I do not put a hot bath towel on my head, all around my head to calm myself …

In order to mask the tinnitus, Lucie opened the windows on to the street to hear the constant noises of the cars. When she had to stay at home, she turned on the radio as loud as she could, or walked or cycled just to think about something else. Lucie is happy to speak of her suffering with her psychologist. She felt people did not understand *what it is to have tinnitus* all day long and for the rest of their life and they ignored her suffering, because they were not able to hear it. When she talks about her problem with others, they answer with their own physical troubles. Lucie was asking for help, but her family circle was not concerned by what had happened to her.

Eventually, Lucie moved to a smaller apartment, following the advice of her sister. She wanted to leave the old apartment in which she felt sick and thought that the longer distance to the radio relays would perhaps lead to improvements in her health. But in her new apartment, she noticed the silence around her: no more cars passing through the street, no liveliness from a familiar neighbourhood. After weeks of improvement her condition worsened again. Her tinnitus became louder and more intrusive. Lucie was exhausted and weary of her loneliness and suffering. *The tinnitus does not bother me any more:* she said weeks before, *because I can control him: I forget him*. Now she is upset by yet another hospitalisation that her psychiatrist recommends. The last time Lucie came out from the hospital her condition was worsened for no reason. She *would like to understand why she can't be happy any more*, since she has always been able to manage life.

While the psychotherapy progressed, Lucie was invited to speak about her close relatives and her relationships with her neighbours. She remembered that she was not able to follow through with suicide on the bridge, when her grandson came to mind. She had not had any news from him for over a year (the time before her hospitalisation). Thinking of her grandson makes her anxious. He is homeless and has been roaming the streets for months. He is unemployed and associates with young people who seem to be a bad influence on him. She knows her grandson very well. He can be driven by others to do things he does not want to do, for instance, to buy alcohol or drugs.

I am always afraid that something will happen to him with the police force. I think of him all the time. No day goes by without me worrying about him. Not a day. It is exhausting to think everyday about the same thing.

After a brief silence Lucie adds: *But above all there is the tinnitus, which is always there … This is also exhausting. It is always there* … Sometimes she has two or three peaceful days, but in the evening the tinnitus is once again louder and annoying. Lucie tries to forget her worries by getting out for walks as often as she can. She explains in the following words her daily struggle against fears and concerns: *If I go outside, then I think less about all of it. But if I pass the youth in the street, if they talk about an arrest on television, I become afraid that something happened … I don't know*. After her son's divorce, the relationship with his son and her grandson became more complicated. His father put him in a youth rehabilitation centre. The son will never forgive his father for that. Lucie has always been close to her grandson, as she says: *He is everything for me, I recognize myself in him*. Lucie suffers not being able to see him anymore, not to hear him and to know about his life. *My whole body hurts when I think of him, it is inside of me* … They had their last conversation on the phone a few months before her hospitalisation. He hung up suddenly after she said that he must find a job and live decently. She was really furious about his way of life roaming the streets, wasting his life with bad people and risking being driven to behaviours that were not like him. She is annoyed with herself about that. She worried that she should not have talked to him in this way. His way of living should not be of concern to her. He does not want to see her anymore, neither his father. *I don't hear him* … she says. *I wish so much that he calls me, to hear his voice again*. When he was a child she spent every holiday with him and his brother. Now she is left alone, and her family hardly cares for her.

It hurts me. That is the reason why I had a depression. Something happened inside me that I did not understand. What it is that has made me fall over like this … Something in me betrayed me. My body … it must be something that has triggered all of this …

### Beyond the medical condition

The medical explanations about tinnitus left Lucie puzzled, because she noticed something else when she came back from the hospital. She regrets the reaction of her family and neighbours: *People don't believe the satellite dishes' influence … Neither do you?* She is not certain but remembered that a third radio relay was set up near her apartment a short time before the onset of tinnitus. It happened during school holidays and on the weekend, Lucie reports: *like* *a feeling of waves which had seized (her). Something very, very strong* … She has her own opinion about feelings, related to the radio relay, but no one had taken her seriously: *It was during the holidays so there might be much more traffic on the relay, because people call each other more then*. She moved to another apartment, far away from the relay, but tinnitus was still in her ears.

Talking about her previous apartment, Lucie remembers: *I heard that they were struggling upstairs, and they made a lot of noises, they banged on the floor like that* (she mimics a brisk movement while she talks about the noises).

I had an anxiety attack. I started to panic! I could not stay at home. I was too scared. All that noise, they were making, it was awful. I walked out of my apartment during the night; I couldn't manage to bear it any more. I was very scared. I stayed near my building, under the streetlights not to be too scared. I walked out at one o'clock and came back when I was too cold – it was winter this time. It lasted for three months.

Lucie was exhausted from months of irrepressible anguish when she called the gendarmerie and the firemen, the same day her daughter gave her consent for a psychiatric hospitalisation. She needed to be helped, and have someone stretch out a hand towards her. *But they told me that it was me inventing all of it. It came from my imagination, my head*. She finally told the psychotherapist about the noises:

They were making traffic of credit cards and cheques. I heard their machine that made a lot of noise. They only run their machine during the night and not in the day, isn't it strange? They were three upstairs. I saw them come in.

There were no other witnesses of this traffic in the building, only Lucie talked about it. Here the psychotherapist remembered Lucie's fear for her grandson and the risk that he would be forced to be part of drug trafficking, when she continued to talk about traffic making. Coming back from the hospital Lucie thought that the police had arrested the three traffickers, because there was no noise anymore. Another man was then arrested, living near her new flat: *He was trafficking false money … Isn't it really strange? For people like me who just want to be happy?* Many times Lucie signed cheques for her grandson, and for several months she had lodged him in her former apartment. She was hoping that the money would help him to find a job, but he spent it on drugs and alcohol, and broke the bonds between them. Each time she heard a police's sirens, she worried for her grandson.

Lucie had her own reason to fear that something worse would happen to her grandson. She never talked about the reason behind this fear with her daughter and son, despite her family doctor advising her to do so. He was the only one who knew her story. The fear that something irreversible would happen to her grandson leading to him being arrested by the police, came from a dramatic experience Lucie had when she was 22 years old, almost the same age as her grandson. However, in her life the police had never been brought in, because when she had been raped she had never reported the crime. She did not complain then, as no one talked about those things 50 years ago … She kept silent about this event until now, talking about her grandson to a psychologist who was just a little bit older than the grandson. At the time of the rape Lucie was looking for a job and her assailant informed her about a job that was available. She trusted him. *I can remember the frame (where it happened), but what happened there I can't* …*—but that is nothing compared to what I am suffering now* … she added. She did not mention the rape again during the remaining psychotherapy sessions. Lucie kept her worries about her grandson hidden during the months after his disappearance just as she did after the traumatic event she experienced 50 years ago. For the last interview on the telephone, a few days after this recall, she wanted to tell the psychotherapist *something extraordinary*. One morning she woke up with *the feeling that* [she] *missed something*, but she could not say *what was missing*. She finally realized that she did not hear her tinnitus. Lucie was so happy and remembered her thought then: *this is it, they left me quiet, they finally left*. During the whole day she did not hear them. *But in the evening they came back, softly*. Now she could tolerate her tinnitus, and thanked the psychotherapist for what he did for her: *I found someone I could talk to about what happened to me. I will not forget it*.

### Narrative breakdown and psychotherapy

According to Heidegger (1962), intense anxiety can be characterized as an experience of disintegration that, at times, can control a person's life in a way that includes all areas in which this person is involved and life becomes unmanageable. In the present communication, Lucie's whole life had turned into a scenario that she could not manage on her own. Being hospitalized for psychiatric treatment did not have any effect on the disintegration she experienced and disclosed in the psychotherapy sessions. The only objective for Lucie was to get through the day and the night, waiting for relief and order, instead of chaos. Among others, Polkinghorne (1988) has described the mental illness as, in part, to suffering from an "incoherent story" and a great number of authors have defined psychotherapy as an "exercise in story repair" (see also, Schafer, 1992; Spense, 1982). In line with this, Spense (1982) argues that the focus of psychotherapy is on meanings, which are communicated and changed through language. The psychotherapist works with the construction of meanings through narratives rather than discovering meanings in the minds of the client (Polkinghorne, 1988); that is, the therapist plays a role as a collaborator in the production of the narrative.

The structure of the narrative expressed in Lucie's suffering can be elicited in a temporal dimension. The beginning of the narrative focused on tinnitus as exhausting sounds that she was unable to handle by herself. Her endeavour to express her needs to be heard was in vain. Through the psychotherapist's trust in her story of despair, a progression in the narrative could take place. The tinnitus became less annoying. Lucie wanted to convince the psychotherapist that she was able to adjust to it (i.e., that she deserved his support). A parallel narrative including her grandson was then associated to a regression in her suffering partly as a justification of her inability to keep her improvements in tinnitus habituation (perceived expectation of her interlocutor from a Tinnitus clinic). The middle of the narrative structure can be recognized when Lucie told the psychotherapist about her grandson's present situation. Regression in the suffering associated to tinnitus increased, without any possible justification related to her fear for the grandson. Lucie did not know what else could improve her habituation to tinnitus, besides reassuring news about her grandson. She had, however, noticed that no change in her annoyance was associated with this kind of news. She questioned the psychotherapist's support, for example, did she really deserve it, as she was unable to adapt to the tinnitus. Her failure to tolerate tinnitus, she feared, would lead to another dreadful psychiatric hospitalisation. The ending of the narrative structure is the focus on the psychotherapist's disclaimer regarding the failure of habituation to tinnitus and Lucie's experience of worthlessness. She finally accepted belief in herself. The noises she heard and feared upstairs in her apartment were enlightened in a memory that Lucie did not want, or was unable, to share during months of psychotherapy. Finally, at the end, the hallucinations she was unable to share with anyone were disclosed with fear and anguish. The rape that she eventually recalled, after 50 years, can be considered as the overcoming of a deception regarding others attitude towards her narrative; that is, Lucie managed to tell the truth about the fear she had for her grandson, to another who ignored it, like her close relatives during all these years. Then tinnitus in her narrative became a tolerable symptom from a difficult period in her life that she managed to get through.

### Psychodynamic insights on emotional drives

Lucie talks about severe alienation, as she is no longer able to recognize herself being in a strange situation. She had felt strong and independent, managing to live a life on her own for many years. However, within a year and a half she lost an important object relation when her grandson detached himself from her. Lucie's role, as a giving and needed grandmother, changed into grief and feelings of deprivation. Her closest family bonds became fragile and she felt alone, overwhelmed with despair when telling the daughter and son about all the noises that she experiences. The psychotherapy allowed Lucie to talk not only about her tinnitus, but also about the individual and historical background to her suffering. Insights on her relationship with her grandson and her own youth enlightened her emotional drives and difficulty to habituate to tinnitus. Lucie managed to recall in psychotherapy a rape when she was young, that is, about the same age as her grandson. Nobody in her family knew about this traumatic incident, because Lucie never talked about it—she had repressed and forgotten about it. When tinnitus occurred, after months of irrepressible anguish, Lucie was faced with ignorance from the people she tried to confine. Her concern for her grandson led to an increase in anxiety, and was related to what she feared the most: something terrible could happen to him, just like it did to her, and she could not find words to prevent it. Weeks after the beginning of psychotherapy, tinnitus became less of a topic of complaint, due to the deeper conflict that had been recognized and worked through, leading to enlightening of her unconscious emotional drives.

The *threat* of breakdown that Lucie reported in her narrative changed during the psychotherapy process into a progressively more personal reality. Unbearable sounds in her head (tinnitus) were the first expressive form of her exhaustion and were being ignored by significant others. A second form of expression was the fear regarding her grandson's relationships and the bad influences that she was unable to prevent. This fear of breakdown was also associated to an event that *could* *happen* to him in an uncertain future. A third form was recalled by the noises made by traffickers fighting upstairs. The anguish expressed in her narrative lead finally to the rape she suffered, as Lucie already unwittingly disclosed through a "slip of the tongue". She remembered: "they *struggled* upstairs …" which she said in order to express what she heard (and not: they were fighting upstairs, a word which is closer in French than in English; ils se débattaient—ils se battaient). Finally, the rape is recalled as a traumatic event in her history but without any associated emotion—an empty frame without content—*a place where something* *happened*. The fear of breakdown, as written by Winnicott (1989), is the inner perception of a trauma that the psyche is unable to integrate as a past event, and therefore perceive as a breakdown *to come*. In Lucie's narrative, the fate of her grandson helped her to present the trauma to another (the psychotherapist).

As the psychotherapeutic process progresses, the patient often focuses her narrative on her relationships to others and her emotions related to a suffering that nobody knows and therefore cannot take into account. In our clinical experience, the patient is seldom indifferent to the others' incomprehension of her very unusual and sometimes unbearable situation (Erlandsson, 2000). Repeated deception regarding others' attitude toward the complaint of tinnitus can lead the psychotherapist to deeper emotional drives that the patient cannot express more directly. We can see here that Lucie repeatedly expresses regret through her narrative: nobody *believed* her when she tried to explain what she feared. Others challenged all the noises upstairs, the tinnitus that no one else could hear, and Lucie's belief that the satellite dishes had influenced her condition. Would people have believed her, if she had decided to disclose the rape that she suffered as a young woman? The reactions she met from her family and neighbours disappointed her. As for the noises upstairs in her apartment, her telling them was useless. Fifty years ago she was convinced that no one would have listened to the complaint of a raped young woman. Looking back on the psychotherapeutic sessions it should be noted that the social dimension of Lucie's suffering appears to be the unifying thread of her narrative, which finally moves her anguish to a deep and convincing reasoning.

## Clinical discussion

We emphasize strongly that the patient's physical suffering is not dismissed in the present approach rather that we have tried to explain that physical suffering can awake forgotten memories of a hidden trauma. This is not unusual when someone is affected by a long-standing tinnitus. Carrying the burden of tinnitus is challenging and troublesome if you also are trying to balance memories of traumatic events by keeping them under the surface (Fagelson, 2007). The distinction between the symptom phenomena and the suffering needs to be clarified. No one talks about it in the same way—it is a subjective phenomenon. What is physical suffering? You can suffer from pain in your body but this experience is not the same as someone else's experience who also suffers from pain.

For a long time, psychological literature on tinnitus has associated annoying tinnitus, to a significant level, with anxiety (e.g., Erlandsson & Persson, 2006; Hesser & Andersson, 2009; Wilson & Henry, 2000). However, in the scope of the habituation model, such anxiety is to be considered as a distorted and maladaptive perception of tinnitus (Hallam & McKenna, 2006; Sweetow, 2000). Therefore, the model states that this emotion should be corrected with educational and/or behavioural techniques. In the psychodynamic scope, when the anxiety appears to be an overreaction to an event or to a physical condition, it is the psychologist's duty to try to understand this anxiety from the patient's viewpoint. Regarding the Lucie case, one can observe that for several weeks, she was unable to explain to herself her emotions related to tinnitus. She found it very difficult to describe to the psychologist the perception of tinnitus, that is, the character of the sounds that she heard but also her fear. Lucie was not prepared to disclose her unusual experience and needed time to find her own words to talk about it. However, as in the case of many of our patients, she had her own viewpoint and the explanations of others did not convince her.

The psychodynamic process helps the clinician to distinguish the inability to express an appropriate reason for a high level of anxiety and the legitimacy of such an emotion (Dauman, 2010). As psychotherapists, we aim to support the patient's thinking and insights of his or her inner world. We do not preclude the intensive emotion if the patient cannot explain it or because it appears inappropriate to the symptom. Insights need the help of authentic, clinical listening which pays attention to the personal history that was interrupted and sometimes stopped by tinnitus. Understanding the suffering from the patient's point of view involves being attentive to her or his self-perception in relation to others, that is, the narrative of the patient to be understood by the clinician. In such a narrative, the patients are telling more than they perceive on their own and are able to question in their narrative.

A general hypothesis (non-specific to tinnitus suffering) supports the psychodynamic approach regarding the narrative, i.e., the patient's words are unwittingly associated to the past with unresolved personal prejudices and suffering (Freud, 1924/2003). The patient expresses to others without a clear consciousness of what she has communicated. Psychodynamic unconscious is basically a repeated expression of suffering, and the patient is not able to overcome this on her own, needing the recognition of another. Human suffering has its roots in early relationships to significant others and demands to be admitted in current ones, for example, during a psychotherapeutic process (Freud, 1924/2003). Pathological relationships deny the expression of the individual suffering and emotional drives, forcing the person to repress her self-perception and her own thinking. The aim of psychotherapy is to build psychological bridges between the patient's speech and the self-perception of his/her emotions and thinking (Shapiro, 2003). The process described below could be designed as follows: repressed suffering decreases with improvement of self-perception, insights on the inner world and the ability to utter it to the psychotherapist.

The word "traffic" that Lucie used several times during the psychotherapy sessions is associated to this kind of reality that people do not want to listen to and agree (concur) to keep silent. Nobody talks about the trafficking, because of the risk of reprisals from a faceless network. Lucie's mother tongue (French) has many names for the word "trafficking". From the radio relay activity to the illicit drug trade she feared that her grandson would fall in to. Freud (1913/1995) supported that patients' speech is unconsciously over-determined to express similar events and thoughts with word associations. Over- determination can be considered the first step of thinking, or searching for a true and singular expression about a suffering that is not bearable. Talking about the increased traffic on the radio relay during school holidays in her narrative, a few days before the onset of tinnitus, Lucie is unconsciously searching for her own reason to be upset for her grandson's future. She finally recalled the rape in her youth, while she was searching for a job—like demanding him to find a job just before he disappears from her life. The psychodynamic psychotherapy gave her the opportunity to be heard with her emotional drives, by a clinician she trusted. Psychoanalysis defines such an emotional process as transference: in her speech the patient is unconsciously searching for another who could listen to what has been neglected in the individual past and demands recognition in the present.

## Method discussion

Using the narrative method demands a careful and discerning balance between the intimacy that is disclosed through the process of psychotherapy and the respect of privacy that we owe to each patient as psychologists. The reconstructed narrative that was made out of what Lucie conveyed took this balance into account from three main aspects: (1) the social identity of Lucie is intentionally poor from a narrative point of view, without any information on her place of living, family and her past employments; (2) the quotations from her could neither be a help for recognition because it deals mainly with the noises (tinnitus and hallucinations) that Lucie was too resigned or frightened to explain to others; (3) nonetheless, what Lucie said about the tinnitus and her grandson allow the reader to listen to her complaints beyond the medical comprehension of the symptom. Such a personal background is seldom taken into account in the literature on tinnitus.

Here we want to emphasize that such a thorough description as in the present case study brings limited knowledge regarding the development of the symptom during the weeks of psychotherapy. The relevance of the narrative approach is elsewhere. We probably would have had more consistent information about this development by the use of statistical assessments however such evaluation would never allow us to enlighten the links between Lucie's narrations of tinnitus and her dramatic past suffering. Only the narrative approach can bring insights on the unconscious roots of a complaint, which is over-determined. To our knowledge, such an approach of tinnitus suffering, which is intentionally focused on the patient's discourse addressed to the clinician, has never been described before in the literature. Instead the patients' emotions are presented from a quantitative view (i.e., abnormal levels of anxiety and depression) without respect for the subjective and personal contexts of these emotions. We believe that such an approach has limited contribution to the understanding of tinnitus annoyance, because emotions are intrinsically personal and historical.

We have chosen to focus the narrative on the speech of the patient, in order to make links to her personal history. The complexity of this perspective (i.e., to use the patient's quotations as the main material for the narrative analysis) demanded the exclusion of yet another perspective, that is, that of the psychotherapist's countertransference reactions and what the effects are on the patient's emotions. However, we do believe that the transference–countertransference issue is a very important part of the therapeutic process, and that it should be the main concern in another study using a psychodynamic approach with patients suffering from tinnitus. At last, this case study has its intrinsic limitations compared to, for example, the standards of the experimental research. Therefore, it cannot be proposed as a general conception of the suffering from tinnitus, but rather a consistent approach of a reality that demands to be tried and tested by other psychologists involved in the field.

## Conclusion

In the present communication, the focus is on the narrative of the patient, in order to confirm how the process of psychotherapy can contribute to a clarification of the tinnitus complex. The narrative approach is not a value-free, neutral, or amoral science (Crossley, 2000); it deals with subjectivity and personal meaning. The same can be said about psychoanalysis. Tinnitus is also a subjective phenomenon; a perceived inner noise that no one else can hear. The meaning attached to the tinnitus can be highly emotional and a substitute for inner turmoil leading to existential anxiety. Pointing to the concept of projection and identification, one can refer to Kennedy (1953) who suggested with the notion that the patient might project her imagination into the tinnitus sound in a similar way as in a Rorschach test. Finally, we would like to stress the importance of capturing not only the patients' reactions to tinnitus and their general level of stress, but also the search for the key to the unrelieved trauma.
